# Relation between electrical properties of aerosol-deposited BaTiO_3_ thin films and their mechanical hardness measured by nano-indentation

**DOI:** 10.1186/1556-276X-7-264

**Published:** 2012-05-22

**Authors:** Hong-Ki Kim, Jong-Min Oh, Soo In Kim, Hyung-Jun Kim, Chang Woo Lee, Song-Min Nam

**Affiliations:** 1 Department of Electronic Materials Engineering, Kwangwoon University, 447-1, Wolgye-dong, Nowon-gu, Seoul, 139-701, South Korea; 2 Department of Nano and Electronic Physics, Kookmin University, 861-1, Jeongnung-dong, Seongbuk-gu, Seoul, 136-702, South Korea

**Keywords:** BaTiO_3_, thin film, aerosol deposition, nano-indentation, leakage current

## Abstract

To achieve a high capacitance density for embedded decoupling capacitor applications, the aerosol deposition (AD) process was applied as a thin film deposition process. BaTiO_3_ films were fabricated on Cu substrates by the AD process at room temperature, and the film thickness was reduced to confirm the limit of the critical minimum thickness for dielectric properties. As a result, the BaTiO_3_ thin films that were less than 1-μm thick showed unstable electric properties owing to their high leakage currents. Therefore, to overcome this problem, the causes of the high leakage currents were investigated. In this study, it was confirmed that by comparing BaTiO_3_ thin films on Cu substrates with those on stainless steels (SUS) substrates, macroscopic defects and rough interfaces between films and substrates influence the leakage currents. Moreover, based on the deposition mechanism of the AD process, it was considered that the BaTiO_3_ thin films on Cu substrates with thicknesses of less than 1 μm are formed with chinks and weak particle-to-particle bonding, giving rise to leakage currents. In order to confirm the relation between the above-mentioned surface morphologies and the dielectric behavior, the hardness of BaTiO_3_ films on Cu and SUS substrates was investigated by nano-indentation. Consequently, we proposed that the chinks and weak particle-to-particle bonding in the BaTiO_3_ thin films with thicknesses of less than 0.5 μm on Cu substrates could be the main cause of the high leakage currents.

## **Background**

The ever increasing functionality, portability, and switching frequencies of consumer electronics, which cause parasitic inductance effects such as electromagnetic interference (EMI) and simultaneous switching noise (SSN) [1,2], are putting tremendous pressure on designers and manufacturers to pack more circuitry into a smaller space at the same time as suppressing SSN and EMI. Because of these demands, the three-dimensional integration of passive components should be realized instead of relying on existing surface-mounted passive components, i.e., two-dimensional integration, owing to the occupation of space by the passives over the entire printed circuit board area [3]. Among the passive components requiring three-dimensional integration, the development of an embedded decoupling capacitor is particularly important for miniaturization and the suppression of EMI and SSN [4,5].

As candidates for an embedded decoupling capacitor, there are low-temperature co-fired ceramics (LTCCs), polymer composites, films deposited by sputtering, and so on [6]. However, LTCCs still require high-temperature processes around 850°C, which pose a critical problem for the embedding of passive components [7], and polymer composites have poor dielectric properties [8]. Also, ferroelectric films deposited by sputtering must be annealed at a high temperature in order to achieve the desired crystalline phase and orientation [6]. Therefore, to overcome the processing problems, a new technological approach has been attempted using an aerosol deposition (AD) process that enables us to fabricate dense ceramic thick films at room temperature. Although the AD process was invented for the growth of ceramic thick films [9], we applied the AD process as a thin film fabrication process to achieve a high capacitance density of over 1,000 nF/cm^2^[10].

In our previous research, to produce a high capacitance density, BaTiO_3_ was deposited on Cu substrates by the AD process at room temperature, and the film thickness was reduced to confirm the critical minimum thickness for dielectric properties. Consequently, all BaTiO_3_ thin films that were less than 0.5-μm thick showed short-circuit characteristics owing to high leakage currents, while the BaTiO_3_ thin films that were 0.5- to 1-μm thick had only partial dielectric properties, which was an obstacle in achieving a satisfactory thin film process using the AD process [11-13]. However, the BaTiO_3_ films that were more than 1-μm thick exhibited a dense morphology from which defects were markedly absent, which was considered to be due to the rigidity of the surfaces of the hard ceramic BaTiO_3_ films. From the above consideration, in order to clarify the role of substrate hardness in growing the deposited films, BaTiO_3_ films were fabricated on stainless steel (SUS) substrates which were harder than the Cu substrates. As a result, the BaTiO_3_ films on SUS substrates had a lower critical minimum thickness of 0.2 μm, a fewer macroscopic defects, and a lower interface roughness than the BaTiO_3_ films on Cu substrates. Thus, we discussed that the high leakage currents were related to macroscopic defects and a rough interface [14].

In this study, in order to reveal the influence of the macroscopic defects and the rough interface on the leakage currents, a current image of the macroscopic defects was obtained by conductive atomic force microscopy (C-AFM) analysis, and the roughness of the interface between the films and the substrates was also observed by the focused ion beam (FIB) technique. Moreover, based on the deposition mechanism of the AD process, it was considered that BaTiO_3_ thin films of less than 1 μm in thickness are formed on Cu substrates with chinks and weak particle-to-particle bonding, and these surface morphologies are related to the presence of leakage currents. Therefore, the hardness, which depended on the surface morphologies of the BaTiO_3_ films on Cu and SUS substrates, was measured by nano-indentation, and the relation between the hardness and the electrical properties of the BaTiO_3_ films on Cu and SUS substrates was investigated to identify another cause of the high leakage currents.

## **Methods**

The AD process is based on shock-loading solidification due to the impact of ceramic particles. During the AD process, fine ceramic particles are accelerated by gas flow in the nozzle up to a velocity of several hundred meters per second and are sprayed onto the substrates. The details of the AD process apparatus can be referred to elsewhere [15]. BaTiO_3_ films were deposited by the AD process using a commercial BaTiO_3_ powder (BT-045 J, Samsung Fine Chemicals Co., Ltd., Ulsan, South Korea) with a particle size of 0.45 μm as a starting powder. The particles were aerosolized in an aerosol chamber and transported into a deposition chamber using He gas at a flow rate of 5 L/min. The transported BaTiO_3_ powder was continuously ejected through the nozzle and deposited onto the Cu and SUS substrates, respectively. The orifice size of the nozzle, the deposition area, the distance between the nozzle and the substrate, the working pressure, and the deposition time were 10 × 0.4 mm^2^, 10 × 10 mm^2^, 10 mm, 3.4 Torr, and 1 to 10 min, respectively.

The dielectric properties of the BaTiO_3_ films with thicknesses of 0.1 to 2.2 μm on Cu and SUS substrates were measured using an impedance analyzer (HP 4194A, Agilent Technologies, Inc., Santa Clara, CA, USA) and their dielectric behaviors were confirmed. The crystallinity and crystallite size of the BaTiO_3_ films on the Cu and SUS substrates were analyzed using an X-ray diffractometer (XRD; X’Pert PRO, PANalytical, Almelo, The Netherlands). Moreover, in order to confirm the causes of high leakage currents, the roughness values of the interfaces between deposited films and substrates were observed through a FIB system (Helios 600i, FEI, Hillsboro, OR, USA), and the surface morphology and current images were observed by C-AFM (SPM 9600, Shimadzu Corporation, Kyoto, Japan). During C-AFM analysis, a voltage of 5 V was applied. In addition, a field-emission scanning electron microscopy (S-4700, Hitachi, Ltd., Chiyoda-ku, Japan) analysis was conducted, and the hardness of the BaTiO_3_ films was investigated by nano-indentation (TriboIndenter, Hysitron, Inc., Eden Prairie, MN, USA) which has been established as a powerful method to characterize the near-surface mechanical properties of materials. For the measurement of the hardness of the BaTiO_3_ films, a Berkovich tip was used, the diameter of which was about 100 nm, which can avoid the influence of the surface roughness of the BaTiO_3_ films on the measured data. The details of the nano-indentation can be referred to elsewhere [16-20].

## **Results and discussions**

### **Influence of rough interface and macroscopic defects on leakage currents**

In our previous research, BaTiO_3_ films with thicknesses of 0.1 to 3.0 μm were fabricated on Cu substrates by an AD process at room temperature and compared with BaTiO_3_ films on SUS substrates. As a result, all BaTiO_3_ thin films with a thickness of less than 0.5 μm showed short-circuit characteristics owing to high leakage currents, and BaTiO_3_ thin films of 0.5 to 1 μm in thickness had partial dielectric properties [11-13]. In addition, the BaTiO_3_ thin films on Cu substrates exhibited rough interfaces between the films and the substrates as well as macroscopic defects such as pores and not-fully-crushed particles. On the other hand, the BaTiO_3_ films on SUS substrates had a lower critical minimum thickness of 0.2 μm, a lower interface roughness, and a fewer macroscopic defects than the BaTiO_3_ films on Cu substrates. From these results, we proposed that the rough interfaces and the macroscopic defects in the BaTiO_3_ thin films with thicknesses of less than 0.5 μm on Cu substrates lead to high leakage currents [14].

In this study, in order to confirm the proposed causes of the high leakage currents, BaTiO_3_ films of 0.1 to 2.2 μm in thickness were fabricated on Cu and SUS substrates by the AD process, and their electrical properties were also confirmed and compared with those of the previous researches. The results for the electrical properties of the BaTiO_3_ films on Cu and SUS substrates are summarized as shown in Table 1. From Table 1, the dielectric properties and the dielectric behaviors of the BaTiO_3_ films on Cu and SUS substrates had almost the same tendencies as those found in previous researches. The critical minimum thicknesses of the BaTiO_3_ films on Cu and SUS substrates were 0.5 and 0.2 μm, respectively. When the thickness of a BaTiO_3_ film was below the critical minimum thickness, the leakage currents abruptly increased, indicating short-circuit characteristics. In addition, BaTiO_3_ films with thicknesses of 0.5 to 1 μm on Cu substrates exhibited partial dielectric behavior, whereas BaTiO_3_ films on SUS substrates exhibited stable dielectric behavior thicknesses of 0.2 μm or more. At the critical minimum thickness of BaTiO_3_ films on Cu substrates, their relative permittivity and loss tangent were 60 and 1.9%, respectively, at 1 MHz. In the case of SUS substrates, they were 59 and 2.1%, respectively, at 1 MHz.

**Table 1:** **Relationship of dielectric properties with substrate type and thickness of BaTiO**_**3**_**films (at 1 MHz)**

**Film thickness (μm)**		**~0.1**	**0.2**	**0.4**	**0.5**	**0.8**	**1.0~**
Cu substrate	*ϵ*_r_	N/A	N/A	N/A	60	69	>71
	tan δ (%)	N/A	N/A	N/A	1.9	1.7	<1
	Capacitance density (nF/cm^2^)	N/A	N/A	N/A	106	76	63<
	Dielectric behavior (%)	*0*	*0*	*0*	*50 to 80*	*50 to 80*	*90 to 100*
SUS substrate	*ϵ*_r_	N/A	59	63	61	60	>65
	tan δ (%)	N/A	2.1	1.9	1.7	2.3	<1.5
	Capacitance density (nF/cm^2^)	N/A	261	139	108	66	<50
	Dielectric behavior (%)	*0*	*90 to 100*	*90 to 100*	*90 to 100*	*90 to 100*	*90 to 100*

**Figure 1:**
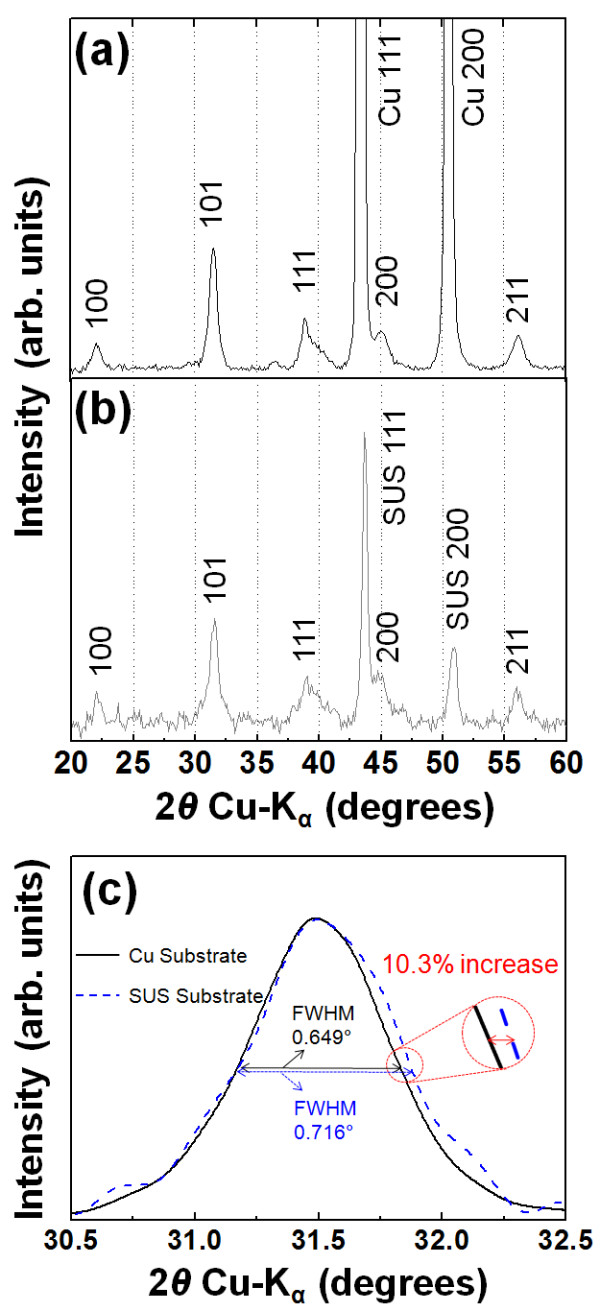
**XRD patterns of the 0.2-μm-thick BaTiO**_**3**_**thin films on Cu and SUS substrates.** (**a**) Cu and (**b**) SUS substrates. (**c**) Cu and SUS substrates between 30.5° and 32.5°.

In order to determine the proposed causes of high leakage currents in the previous researches, BaTiO_3_ thin films with a thickness of 0.2 μm on Cu and SUS substrates were chosen. This choice was made because, although the 0.2-μm-thick BaTiO_3_ thin films on Cu substrates have short-circuit characteristics, those on SUS substrates have a dielectric behavior. The fabricated 0.2-μm-thick BaTiO_3_ thin films on Cu and SUS substrates successively became crystalline as shown in Figure 1a,b. From the XRD patterns shown in Figure 1a,b, it was confirmed that the 0.2-μm-thick BaTiO_3_ thin films on Cu and SUS substrates have a cubic crystal system with a single perovskite phase. The reason for this cubic crystal system of the 0.2-μm-thick BaTiO_3_ thin films on Cu and SUS substrates is their small crystallite size and nonuniform distortion during impaction in the AD process, and it brings about a decrease in the relative permittivity of the BaTiO_3_[21]. However, the 0.2-μm-thick BaTiO_3_ thin films on Cu and SUS substrates exhibited different peak shapes from each other, so that XRD analysis was additionally conducted between 30.5° and 32.5°, which is the region of the 101 main diffraction peaks of the 0.2-μm-thick BaTiO_3_ thin films on Cu and SUS substrates, as shown in Figure 1c. As a result, the XRD patterns confirm that the diffraction peak of the BaTiO_3_ thin films on SUS substrates broadened in comparison with the diffraction peak of those on Cu substrates. This peak broadening was caused by a smaller crystallite size. To support this conclusion, the crystallite sizes were calculated by Scherrer's method. The calculated crystallite sizes of the 0.2-μm-thick BaTiO_3_ thin films on Cu and SUS substrates were 12.6 and 11.3 nm, and the difference in the full width at half maximum was 10.3%. From this result, it was considered that the crystallite size of the 0.2-μm-thick BaTiO_3_ thin films on Cu and SUS substrates was affected by the hardness of the substrates.

**Figure 2:**
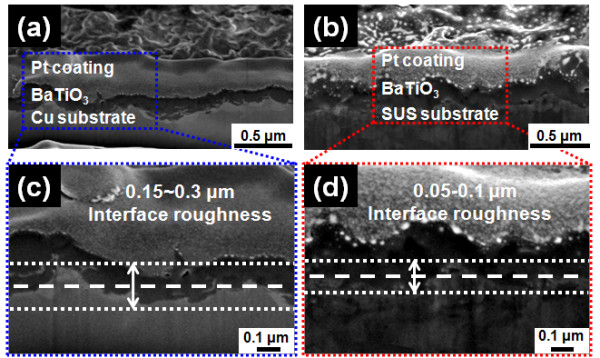
**Cross-sectional SEM micrographs of 0.2-μm-thick BaTiO**_**3**_**thin films on (a) Cu and (b) SUS substrates.** Enlarged cross-sectional SEM micrographs of the films on (**c**) Cu and (**d**) SUS substrates.

**Figure 3:**
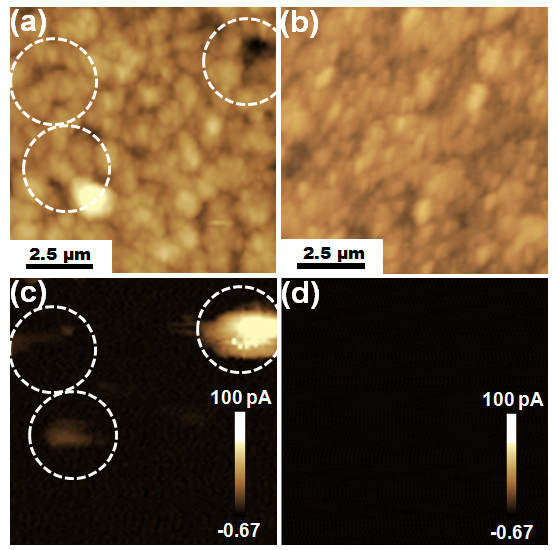
**AFM topographies of 0.2-μm-thick BaTiO**_**3**_**thin films on (a) Cu and (b) SUS substrates.** Current images of the films on (**c**) Cu and (**d**) SUS substrates.

In our previous researches, the roughness of the interfaces between the films and the substrates was observed by peeling BaTiO_3_ thick films of 2 μm in thickness from Cu and SUS substrates to confirm the cause of high leakage currents [14]. As a result, the roughness difference between BaTiO_3_ thick films on Cu substrates and BaTiO_3_ thick films on SUS substrates was confirmed, but the compared films had a higher thickness than the critical minimum thickness. Therefore, in this study, the roughness of the interface between the 0.2-μm-thick BaTiO_3_ thin films and the Cu or SUS substrates was observed by a FIB technique. Figure 2 shows the interfaces of the 0.2-μm-thick BaTiO_3_ thin films with the Cu and SUS substrates. The BaTiO_3_ thin films on Cu substrates had high interface roughness values of 0.15 to 0.3 μm compared with the thickness of the 0.2-μm-thick BaTiO_3_ thin films, as shown in Figure 2a,c. On the other hand, a decreased interface roughness of 0.05 to 0.1 μm was observed for the interfaces between the BaTiO_3_ thin films and SUS substrates, as shown in Figure 2b,d. From these results, we consider that the interface roughness is generated by the impact of the accelerated BaTiO_3_ particles onto the substrate, and thus it is intensified on ductile substrates such as the Cu substrate. Therefore, the BaTiO_3_ thin films on Cu substrates had a higher interface roughness than the BaTiO_3_ thin films on SUS substrates, and an area with a shortened distance between the top of the BaTiO_3_ thin film and the substrate was largely formed. This area can cause a high field concentration, giving rise to high leakage currents, and higher leakage currents are generated for BaTiO_3_ thin films on Cu substrates than for those on SUS substrates [12]. In addition, in order to observe the leakage currents at the macroscopic defects, which are the proposed cause of the leakage currents in our previous researches, the surface morphologies of the 0.2-μm-thick BaTiO_3_ thin films on Cu and SUS substrates were observed by C-AFM analyses. Figure 3a,c shows the AFM and C-AFM images of a 0.2-μm-thick BaTiO_3_ thin film on a Cu substrate, and Figure 3b,d shows the AFM and C-AFM images of a 0.2-μm-thick BaTiO_3_ thin film on an SUS substrate. In the AFM image shown in Figure 3a, it can be seen that macroscopic defects existed on the surfaces of BaTiO_3_ thin films on Cu substrates. Also, high leakage currents were observed in the macroscopic defect region as shown in Figure 3c, whereas the BaTiO_3_ thin films on SUS substrates had no macroscopic defects and no leakage currents, as shown in Figure 3b,d. This result revealed that the leakage currents largely flowed through the macroscopic defects, and thus the macroscopic defects are another cause of the high leakage currents.

### **Surface morphology of BaTiO**_**3**_**films without macroscopic defect areas**

From the above results, it was confirmed that the roughness of the interface and the macroscopic defects have a direct influence on the leakage currents. However, we consider that the causes of the leakage currents are not only the rough interface and the macroscopic defects, but also the chinks and weak particle-to-particle bonding that arise due to the deposition mechanism of the AD process. The bonding of ceramic particles in the AD process is based on shock-loading solidification, resulting from the impact of ceramic particles. The densification of the ceramic films is formed by the continuous impaction of ceramic particles onto the pre-impacted particles or substrate. Therefore, it is considered that the impaction of ceramic particles is not sufficient for the densification of the BaTiO_3_ thin films on Cu substrates, and therefore, these BaTiO_3_ thin films have chinks and weak particle-to-particle bonding. On the other hand, in the case of the BaTiO_3_ thin films on SUS substrates, it is considered that even if the impaction of ceramic particles is not high enough, the ceramic particles are sufficiently fractured to form dense BaTiO_3_ thin films due to the use of harder substrates than Cu substrates. From the calculated crystallite sizes based on the XRD data, the smaller crystallite sizes of the 0.2-μm-thick BaTiO_3_ thin films on SUS substrates compared to those of the 0.2-μm-thick BaTiO_3_ thin films on Cu substrates support the above consideration.

**Figure 4:**
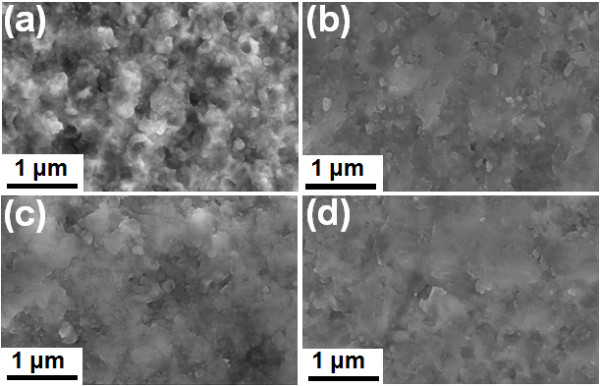
**Surface SEM micrographs.** Surface SEM micrographs of 0.2-μm-thick BaTiO_3_ thin film on (**a**) Cu and (**b**) SUS substrates and 2-μm-thick BaTiO_3_ thick films on (**c**) Cu and (**d**) SUS substrates.

Therefore, scanning electron microscopy (SEM) analysis was conducted on the 0.2-μm-thick BaTiO_3_ thin films on Cu and SUS substrates to observe their surface morphologies. In addition, in order to confirm the results of the above-mentioned densification procedure of the BaTiO_3_ thick films on Cu and SUS substrates, SEM analysis was additionally conducted on 2-μm-thick BaTiO_3_ thick films on Cu and SUS substrates. Figure 4a,b shows the SEM images of the 0.2-μm-thick BaTiO_3_ thin films on Cu and SUS substrates, and Figure 4c,d shows the SEM images of the 2-μm-thick BaTiO_3_ thick films on Cu and SUS substrates. From the SEM images, the 0.2-μm-thick BaTiO_3_ thin films on Cu substrates showed incompact surface morphologies and even chinks. On the other hand, the 0.2-μm-thick BaTiO_3_ thin films on SUS substrates showed dense surface morphologies. In addition, the 2-μm-thick BaTiO_3_ thick films on Cu and SUS also had dense surface morphologies, and the incompact and chink morphologies of the 0.2-μm-thick BaTiO_3_ thin films on Cu substrates disappeared in the 2-μm-thick BaTiO_3_ thick films on Cu substrates. From the above results, it is considered that the weak particle-to-particle bonding due to incompact surface morphologies and chinks in the 0.2-μm-thick BaTiO_3_ thin films on Cu substrates can cause high leakage currents, and the 2-μm-thick BaTiO_3_ thick films on Cu substrates become dense due to the above-mentioned densification procedure of the AD process.

### **Relation between electrical properties and film hardness**

From the surface morphologies of the BaTiO_3_ films on Cu and SUS substrates, it was considered that the chinks and weak particle-to-particle bonding of the BaTiO_3_ thin films bring about an increase in the leakage currents. However, it was difficult to reveal the relation between the surface morphologies and the leakage currents by using only the AFM and SEM analyses. Therefore, in order to exhibit the above-mentioned relation, the hardness of the BaTiO_3_ films with thicknesses of 0.2 to 2.2 μm on Cu and SUS substrates was measured by nano-indentation, and then the tendencies of the hardness and the dielectric behavior of the BaTiO_3_ films were compared.

**Figure 5:**
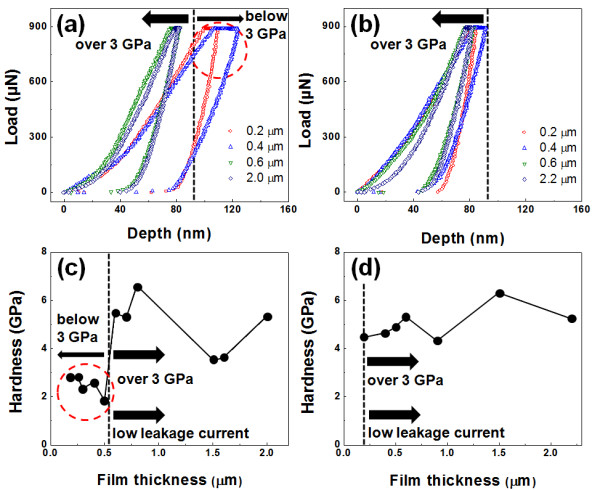
**Load–displacement curve and thickness dependence of surface hardness of BaTiO**_**3**_**films.***P*-*h* curves of BaTiO_3_ films on (**a**) Cu and (**b**) SUS substrates for a load force of 900 μN and the thickness dependence of surface hardness of the films on (**c**) Cu and (**d**) SUS substrates.

Figure 5a,b shows the load versus depth of penetration (*Ph*) data plots of the BaTiO_3_ films on Cu and SUS substrates according to their thickness values, and Figure 5c,d shows calculated hardness data from the *Ph* data plot. In order to properly measure the hardness data of the 0.2-μm-thick BaTiO_3_ thin films on Cu and SUS substrates excluding the effect of the substrate hardness, a load force of 900 μN was used and applied to the BaTiO_3_ films with thicknesses over 0.2 μm on Cu and SUS substrates. From the hardness data, although all the BaTiO_3_ films with thicknesses of less than 0.5 μm on Cu substrates had hardness values of less than 3 GPa, the hardness values of the BaTiO_3_ films with thicknesses of more than 0.5 μm on Cu substrates were increased to more than 3 GPa. On the other hand, the BaTiO_3_ films on SUS substrates had hardness values of more than 3 GPa even for their initial deposition thickness of 0.2 μm. From the above results, it is confirmed that the initial hardness and the surface morphologies of the BaTiO_3_ films are affected by substrate hardness, and the BaTiO_3_ films on Cu substrates become hard and develop dense morphologies as they grow thicker. In the case of the electrical properties of the BaTiO_3_ films on Cu and SUS substrates, the BaTiO_3_ films on Cu substrates exhibited a critical minimum thickness of 0.5 μm, whereas the BaTiO_3_ films on SUS substrates exhibited a dielectric behavior for thicknesses of at least 0.2 μm. To sum up the above results, it was confirmed that the tendency of the hardness of the BaTiO_3_ films on Cu and SUS substrates matched that of the electrical properties. It means that the BaTiO_3_ thin films of less than 0.5 μm in thickness on Cu substrates with chinks and incompact surface morphologies have low hardness values of less than 3 GPa and short-circuit characteristics, whereas the BaTiO_3_ thin films on SUS substrates with dense surface morphologies have high hardness values of more than 3 GPa and a dielectric behavior for thicknesses of 0.2 μm or more. Therefore, we propose that the leakage currents flow through the chinks and the weak particle-to-particle bonding areas of the BaTiO_3_ thin films with thicknesses of less than 0.5 μm on Cu substrates and that this is the main cause of the high leakage currents. In addition, from the increased hardness and the dense surface morphologies of the BaTiO_3_ films with thicknesses of more than 0.5 μm on Cu substrates that develop as the films grow, it is considered that the hammering effect [22], which is a densification procedure that works by continuous impaction of ceramic particles, is an important factor to overcome the causes of high leakage currents. Therefore, to achieve a thin film deposition process based on the AD process, further study of the densification mechanism of the BaTiO_3_ films should be conducted. If the densification mechanism of the BaTiO_3_ films is clarified and the causes of high leakage currents are overcome, the AD process could be successfully applied to embedded decoupling capacitor applications with high capacitance density as a thin film process.

## **Conclusions**

In order to clarify the causes of high leakage currents, BaTiO_3_ films with thicknesses of 0.1 to 2.2 μm were fabricated on Cu and SUS substrates by an AD process at room temperature. The critical minimum thicknesses of the BaTiO_3_ films on Cu and SUS substrates were 0.5- and 0.2-μm thick. From the FIB technique, it was exhibited that the 0.2-μm-thick BaTiO_3_ thin films on Cu substrates had higher interface roughness than the 0.2-μm-thick BaTiO_3_ thin films on SUS substrates, which can cause high field concentrations and therefore give rise to high leakage currents. In addition, it was confirmed from the C-AFM analysis that the leakage currents largely flow through the macroscopic defects. Chinks and incompact surface morphologies of the 0.2-μm-thick BaTiO_3_ thin films on Cu substrates were observed, and the hardness of BaTiO_3_ thin films with thicknesses of less than 0.5 μm on Cu substrates was below 3 GPa. On the other hand, the BaTiO_3_ films with thicknesses over 0.5 μm on Cu substrates and the BaTiO_3_ films of 0.1 to 2.2 μm in thickness exhibited dense surface morphologies and hardness values of more than 3 GPa. It was confirmed that the tendency of the hardness of the BaTiO_3_ films on Cu and SUS substrates matched that of their electrical properties. We explained the above results by concluding that the chinks and weak particle-to-particle bonding bring about leakage currents based on the relation between hardness and the electrical properties.

## **Competing interests**

The authors declare that they have no competing interests.

## **Authors' contributions**

HKK, JMO, SIK, and HJK carried out the aerosol-deposited sample fabrication, measurements, and interpretation of the results. CWL and SMN initiated the idea of working on the present topic and analyzed all experiments. All authors read and approved the final manuscript.
